# Transforming dementia caregiver support with AI-powered social robotics

**DOI:** 10.3389/frobt.2025.1704313

**Published:** 2026-01-27

**Authors:** Tyler Morris, Conor Brown, Xiaopeng Zhao, Linda Nichols, Jennifer Martindale-Adams, Sharon Bowland, Wenjun Zhou

**Affiliations:** 1 University of Tennessee, Knoxville, TN, United States; 2 University of Mississippi, Oxford, MS, United States; 3 University of Tennessee Health Science Center, Memphis, TN, United States; 4 VA Caregiver Center, Department of Veterans Affairs, Memphis, TN, United States

**Keywords:** AI-assisted healthcare, dementia caregiving, human-robot interaction, retrieval augmented generation, social robotics

## Abstract

**Introduction:**

Informal dementia caregivers face significant emotional and physical burdens, yet evidence-based interventions like REACH are often limited by high labor costs and scalability constraints.

**Methods:**

We design a Robot-based Information and Support to Enhance Alzheimer’s Caregiver Health (RISE) system, which uses novel social robotics and generative AI to deliver automated and personalized caregiver training and stress management. RISE uses retrieval-augmented generative AI (RAG-AI) grounded in the verified REACH Caregiver Notebook to ensure content safety and minimize hallucinations. It employs the social robot Pepper to deliver interactive presentations, Q&A sessions, review quizzes, and stress reduction activities. A technical evaluation and a two-phase user evaluation was conducted.

**Results:**

We found that the RISE’s RAG-AI backend achieved 87% correctness and 92% relevancy when compared to ground truth. User feedback indicated strong acceptance, with Likert-scale usability scores ranging from 3.6 to 4.6 out of 5 across all components.

**Discussion:**

These results suggest that combining verifiable AI architectures with embodied social robotics offers a feasible, scalable solution for enhancing caregiver support and wellbeing. Future work could include a larger scale user study involving real informal dementia caregivers.

## 1 Introduction

Alzheimer’s disease and related dementias (ADRD) are progressive and irreversible brain disorders that cause a decline in cognitive abilities, including memory and language, affecting over 6.7 million adults in the United States (Alzheimer’s Association, 2023). Family members often become primary caregivers of persons with ADRD (PwADRD). The Alzheimer’s Association estimates that approximately 11 million family caregivers provide approximately 18.5 billion hours of care for those with ADRD annually (Alzheimer’s Association, 2023).

Compared with caregivers of individuals without dementia, those caring for PwADRD experience double the emotional, financial, and physical stress (Wennberg et al., 2015). In particular, these caregivers are five times more likely to experience work loss (Alzheimer’s Association, 2021). The growing shortage of healthcare professionals trained in geriatrics and dementia care is particularly concerning, given the rapid increase in the population aged 65 and older. This shortage is critical, as 83% of PwADRD receive care at home from family and friends who are unpaid caregivers (Alzheimer’s Association, 2023). Addressing this issue is essential to mitigate potential negative impacts on caregivers’ physical and emotional health.

Interventions targeting informal caregivers have shown substantial benefits for both caregivers and PwADRD. One of the most well-established examples is REACH (Resources for Enhancing Alzheimer’s Caregiver Health), which refers to a series of structured caregiver support interventions designed to reduce stress, improve coping, and enhance caregiving skills. Across the original REACH II intervention and its subsequently adapted versions, REACH is characterized by active, personalized coaching sessions. While some type of structured materials are a central component of the program, REACH is fundamentally a person-delivered behavioral intervention that teaches skills so that caregivers can use materials on their own after the program is over. Despite its strong evidence base, REACH has not been widely disseminated in the community due to cost, labor requirements, and lack of insurance reimbursement (Gitlin et al., 2015). Despite these constraints, REACH is one of the most widely implemented community caregiver interventions (Cannon et al., 2025).

A primary limitation of the currently available socially assistive robotics (SARs) is that the predominant focus is directly on persons with dementia, and often neglects the needs of informal dementia caregivers. Prior work has shown exponential growth in the use of robotics and AI within dementia care as a whole, but most of the research tends to focus on assisting older adults with a diagnosis (Yuan et al., 2022a; b; Kyrarini et al., 2021). While these patient focused interventions are both necessary and well-received (Yuan et al., 2022b), there exists a need to apply these systems to other parts of dementia care. AI and robotics offer the potential to ease the burden on caregivers by helping them manage caregiving challenges and cope with stress (Hazarika, 2020), yet few systems address this directly. We seek to create a system that fills this fundamental gap by targeting informal dementia caregivers, which a previously underserved group. We seek to provide them with custom-tailored informational sessions to address their individual issues and alleviate stress.

Furthermore, while the integration of LLMs has enhanced robot interactivity, widespread adoption is often hindered by the risk of “hallucinations” (i.e., the AI generating incorrect information in response to a user query), which risks the generation of unverified medical advice. Recent works have applied LLMs to social robots to create more engaging, adaptable, and personalized systems (Pinto-Bernal et al., 2025; Yuan et al., 2022b; a). Users have praised this engagement (Otaka et al., 2024; Szondy and Fazekas, 2024), and experts have argued that the resulting human-robot attachment is foundational for any helping relationship (Szondy and Fazekas, 2024). However, reliance on standard generative AI models creates a validity problem; many users do not trust AI content to be generated accurately (Hannig et al., 2025). A highly persuasuve social robot delivering confident, yet false, information poses a heightened risk (Elsheikh et al., 2025), especially in high-stakes scenarios like dementia caregiver training. Prior studies have focused almost entirely on either using social robotics (Thompson et al., 2025) or on the AI enhancement themselves (Pinto-Bernal et al., 2025), yet little work has been done on combining SARs with safe generative AI. Therefore, our work seeks to create a system that not only utilizes the interactivity of SARs, but also leverages novel techniques to use safe and effective LLMs to increase user engagement and acceptance. Leveraging AI-generation and social robots, we design and test the Robot-based Information and Support to Enhance Alzheimer’s Caregiver Health (RISE) system, with the goal of providing scalable, informal dementia caregiver support using a social robot platform. RISE adapts components of evidence-based REACH intervention into a self-guided, AI-powered system that delivers targeted educational content and stress management tools to caregivers without requiring a trained human coach. Designed for informal caregivers, often family members with no formal training, RISE focuses on increasing access to high-quality caregiver education by reducing the burden associated with in-person program delivery. The system aims to meet caregivers where they are: in clinics, homes, or community settings, using intuitive voice-based interaction, familiar caregiving concerns, and tailored support content. Its design reflects both the structure of established caregiving curricula and the emerging potential of conversational AI and social robotics to deliver health-related guidance in a naturalistic, empathetic format.

The RISE system stands apart from existing literature by combining the engagement of SARs with the safety of retrieval augmented generation (RAG). As summarized in Table 1 below, RISE addresses the two previously identified gaps: it shifts the user focus from patient to caregiver, and shifts the technology from standard generative AI to verified RAG architectures. By using RAG backed by a certified database, RISE ensures all generated content is traceable to valid sources, minimizing the chance of hallucination found in many AI systems (Hannig et al., 2025). Consequently, RISE utilizes advanced, AI-enhanced social robotic technologies to foster engagement while reducing the previously associated risks of using generative AI.

**TABLE 1:** Comparison of RISE versus existing literature in AI-enhanced dementia care.

Feature	Existing literature/Standard SARs	RISE system (Proposed)
Primary Target User	Persons with Dementia (Patients) (Yuan et al., 2022b)	Informal Dementia Caregivers
Primary Goal	Emotional wellbeing and companionship Kyrarini et al. (2021)	Caregiver education, stress reduction, and training
AI Architecture	Standard Generative LLMs Pinto-Bernal et al. (2025)	Retrieval Augmented Generation (RAG)
Information Source	Pre-trained model data (prone to hallucinations) Hannig et al. (2025)	Certified, closed-domain database
Reliability Strategy	Relies on user trust in the robot’s persona Szondy and Fazekas (2024)	Relies on verifiable, retrieved evidence to prevent misinformation

In this paper, we describe the design and development of the RISE system and report the findings of a preliminary evaluation. We detail the user-centered design to build the system’s architecture, including its modular session structure, knowledge retrieval components, and speech interface, and explain how the content from the REACH Caregiver Notebook was adapted into an interactive, robot-delivered format. We then present the results of a two-phase evaluation: first, usability testing with technically literate users to identify and refine system issues in a low-risk environment, and second, expert review by dementia caregiving professionals to assess the system’s fidelity, clinical relevance, and real-world applicability. Together, these contributions establish the feasibility of using AI-powered social robots to support informal dementia caregivers and offer a foundation for future in-home and clinical deployment studies.

## 2 Resources for enhancing Alzheimer’s caregivers health

The Resources for Enhancing Alzheimer’s Caregivers Health (REACH) program is a well-established, evidence-based intervention that provides structured support for family caregivers of PwADRD. The original REACH II intervention demonstrated strong evidence for improving caregiver outcomes through structured behavioral support (Belle et al., 2006; Elliott et al., 2010), and was adapted into several subsequent versions, such as REACH Community and REACH VA. Researchers created the Dementia Caregiver Notebook to provide training materials and document important domain knowledge in caregiving for PwADRD.

### 2.1 Implementations of caregiver training

Building on extensive clinical research, REACH combines education, skill training, and stress management to improve caregiver wellbeing and reduce burden. It is typically delivered by trained interventionists using standardized materials and involves personalized coaching across multiple sessions. The foundational REACH strategies align with broader recommendations for scaling dementia caregiver interventions, such as those of National Academies of Sciences and Medicine (2016) and Gitlin et al. (2015), which emphasize flexibility, cultural relevance, and caregiver empowerment.

The original REACH II intervention involved structured, multicomponent support delivered over several sessions by trained personnel, and was shown to improve caregiver health outcomes (Belle et al., 2006; Elliott et al., 2010). The REACH II framework was built on earlier caregiver intervention studies (Wisniewski et al., 2003) that emphasized personalized coping strategies and behavior management. This program was translated into other versions such as REACH OUT (Burgio et al., 2009) and Community REACH (Czaja et al., 2018).

REACH VA adapted REACH II for delivery within the U.S. Department of Veterans Affairs, which demonstrated how the intervention could be scaled into healthcare systems (Nichols et al., 2016). The REACH VA streamlines REACH II interventions into four structured sessions delivered by trained staff (Nichols et al., 2016). Subsequent studies have confirmed that this adaptation reduces caregiver stress and even healthcare costs (Nichols et al., 2017).

Further adaptations, including REACH Community and tribal implementations, enabled delivery by community-based staff in underserved settings (Martindale-Adams et al., 2017). REACH Community and REACH OUT further extended the intervention by using lay coaches to reach underserved, rural, and racially diverse populations (Martindale-Adams et al., 2017; Burgio et al., 2009). Community-based adaptations have been studied for feasibility and fidelity in real-world care settings (Czaja et al., 2018), and efforts to adapt content for American Indian and Alaska Native communities have also been explored (Martindale-Adams et al., 2017).

Traditionally, dementia caregiver education in programs like REACH has been delivered through in-person or telephonic sessions led by trained interventionists. These sessions typically occur over a 4-month period and include information and education, support, and skills building, including the three critical skills of cognitive reframing, stress management techniques, and problem-solving tailored to each caregiver’s needs. Interventionists provide individualized coaching and use printed materials to guide discussions, practice, and reinforce their skills.

While this model has been shown to reduce caregiver stress and improve outcomes, it is labor-intensive and resource-dependent, requiring significant time from skilled professionals and multiple sessions to complete. Furthermore, despite its proven effectiveness, REACH and similar interventions are not currently reimbursed by Medicare or most commercial insurers, making it difficult to implement and sustain at scale in real-world settings. As a result, access to this kind of structured caregiver support remains limited, although not unknown (Cannon et al., 2025), especially for caregivers outside of large health systems or urban areas.

### 2.2 The Caregiver Notebook

The risk assessment and caregiver notebooks were developed as core components of REACH II, REACH VA, and REACH Community interventions. These materials were created through partnerships with subject matter experts, healthcare professionals, and dementia caregivers and were tailored to address common caregiving challenges. For this project, the Risk Assessment included three questions about caregiver wellbeing and 23 questions about the presence or absence of care recipient behavioral issues (e.g., aggression, wandering).

The risk assessment is a structured screening tool developed to identify common challenges faced by informal dementia caregivers. It includes 23 yes/no items that cover behavioral issues for the care recipient (e.g., aggression, wandering). The tool was originally designed for use in the REACH II program and has since been modified and implemented by numerous community agencies that support dementia caregivers. Risk assessment helps inform decisions regarding which support resources or educational interventions may be the most needed. A list of these 23 questions can be found in Supplementary Appendix A1.

The Caregiver Notebooks provide caregivers with practical strategies for managing dementia-related behaviors, stress, and self-care. These educational materials were based on the REACH II intervention. There are at least two versions of this notebook: REACH Community and REACH VA Dementia. For the purposes of RISE, we use the REACH Community version of the notebook, as it is suggested by our collaborators and domain experts.

The Caregiver Notebook is a 300-page document that includes a general module on Alzheimer’s disease, multiple stress management activities, such as breathing, stretching, guided imagery, and 48 modules on the management of common behavioral and safety challenges, caregiver stress, self-care, and coping. These 48 modules may be further categorized into 30 behavioral issues, such as bathing, confusion, and wandering, and 18 caregiver coping issues, such as asking for help and managing mood (see Supplementary Appendix A2). Each module contains detailed information on various issues that may arise with a given topic and possible solutions. For example, the “Combativeness” section describes how a PwADRD might be easily agitated by a new environment and how using calming music might alleviate aggression.

The structured, repeatable elements of REACH, such as educational content and caregiver assessments, for behavioral management, are ideal for AI integration. AI systems can help scale REACH education by automating content delivery, personalizing support through data-driven recommendations, and providing continuous engagement via conversational agents, thus making high-quality caregiver support more accessible and sustainable.

## 3 Robot-based information and support (RISE) system design

Using the modules as a framework, the RISE system can provide informative sessions based on various issues that a user might experience. As all the information within the Caregiver Notebook was written and verified by dementia caregiving experts, allowing the AI system to pull information from it balances the adaptability and reliability of information presented to the user.

**FIGURE 1:**
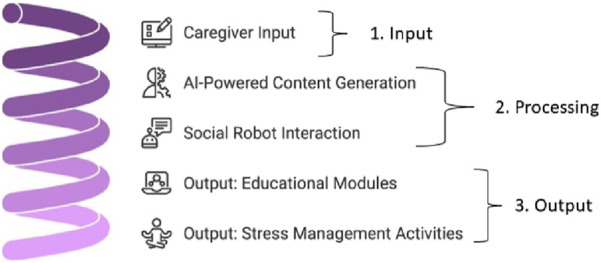
RISE System functionality sequence.

The conceptual framework underlying RISE’s functionality is structured into three key layers: input, processing, and output (see Figure 1). The *input* layer begins with caregiver risk assessment, in which the system collects data to identify stressors and knowledge gaps through a questionnaire. This input is fed into the AI integration process, leveraging generative AI models to create contextually relevant content. The *processing* layer refines the input to provide tailored solutions. AI dynamically personalizes educational content, such as presentations, Q&A sessions, and quizzes to address individual caregiver needs. This content is mediated by Pepper, a humanoid social robot that enhances interactions through empathetic gestures, voice, and visual communication.

The *output* layer delivers tangible support to the caregivers through three key components. First, personalized educational modules provide practical knowledge to address specific caregiving challenges. Second, interactive stress management activities, such as guided breathing exercises and meditation, help caregivers manage their wellbeing. Finally, feedback mechanisms, including quizzes and Q&A sessions, reinforce learning and ensure comprehension, completing the cycle of support offered by RISE. This layered framework ensures a comprehensive, user-centered approach to enhancing caregiver support and education.

### 3.1 The input and output layers

The RISE system can be separated into two parts: the social robot hosting the app (Pepper) and the RISE app that hosts the sessions. The interface was developed using a user-centered design approach, emphasizing accessibility for older adults and caregivers with limited technical experience. Interactions are primarily done through the touchscreen tablet, but some aspects of the RISE app can be controlled with voice. The system prioritizes clarity, natural language interaction, and conversational pacing to reduce the cognitive load and support a comfortable, low-friction experience for users in clinical or home settings.

#### 3.1.1 The social robot pepper

To ensure easy access and engagement with the RISE system, we implemented it through an interactive social robot. Social robots are also known to be more engaging than other forms of technology like mobile apps or webpages (Rosenberg-Kima et al., 2020; Bai et al., 2025).

**FIGURE 2:**
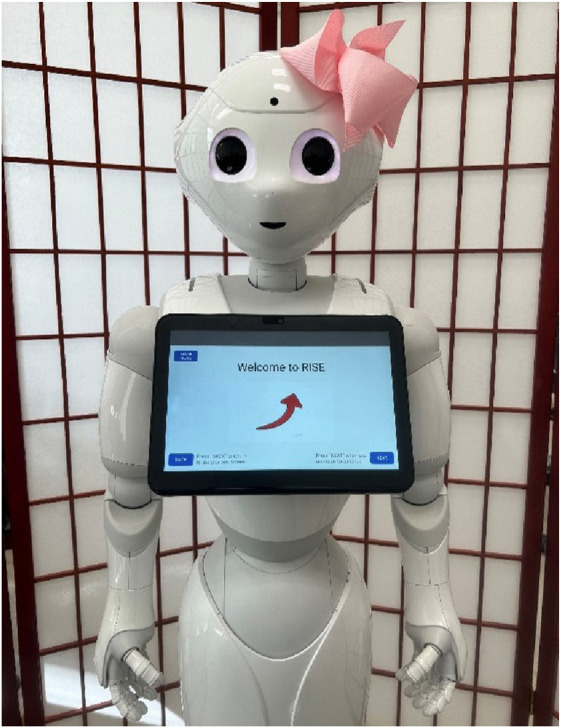
The RISE app on the Pepper Robot.

For this project, we selected the humanoid robot Pepper, illustrated in Figure 2. Pepper features a tablet interface on its chest and can move its arms and body to mimic human movements, speech, and gestures, facilitating interaction with users in various activities. As a social robot, Pepper has been successfully employed in several dementia care applications, with studies indicating positive reception by end users (Yuan et al., 2022a; Yuan et al., 2022b). Due to its effective user engagement, Pepper is an ideal choice for the RISE system.^1^

Applying well-established human-robot interaction principles has been critical to the design of the RISE system. The humanoid robot Pepper exemplifies embodiment and social presence, engaging users through lifelike gestures, conversational cues, and empathetic interactions that foster trust. Personalization and adaptive behavior enable the system to tailor its responses to individual caregiver needs, enhancing satisfaction and perceived value. Transparency in communication ensures that users understand how AI-generated responses are created, building trust in the system’s evidence-based approach; we inform users that AI is used to generate the responses before they interact with RISE. Cognitive load is minimized through concise visual presentations and step-by-step knowledge modules, while stress management activities reinforce user focus and wellbeing. By incorporating these principles, RISE ensures a supportive, accessible, and emotionally intelligent caregiving experience.

#### 3.1.2 The workflow of A RISE session

**FIGURE 3:**
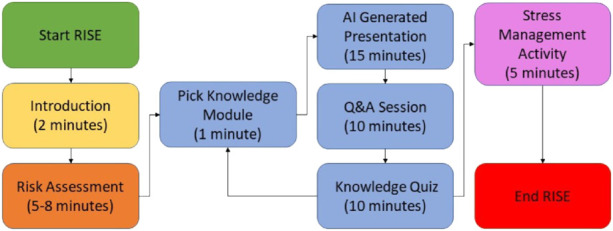
Flowchart of a RISE session.

**FIGURE 4:**
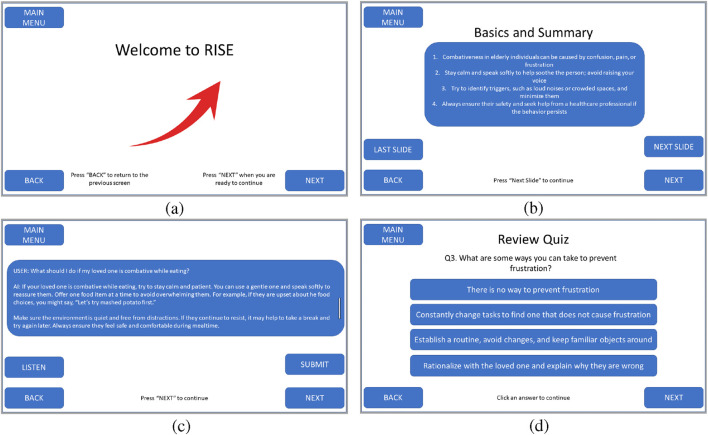
Example screens: **(a)** Introduction, **(b)** Presentation, **(c)** Q&A, and **(d)** Review Quiz.

The app can be further broken down into three parts: 1) the introduction and risk assessment, 2) the knowledge module, and 3) the stress management activity. The app’s workflow is shown in Figure 3, with a few screenshots of key steps shown in Figure 4.

Upon starting the RISE app (green box), users are greeted by a simple introduction screen (yellow box). At this time, they can set the volume for the robot to a comfortable level. A screenshot of the introduction screen is shown in Figure 4a. Caregivers interact through the screen, and speech (i.e., voice-based interaction) is also available in the Q&A section.

To gather information from the user, the robot asks a series of questions representing the Risk Assessment form (orange box). Users respond to these questions one at a time via the tablet interface on Pepper. Based on their responses, the robot can recommend specific knowledge modules that address the issues the user has identified. Users can either complete a recommended module or pick another one that interests them.

The knowledge module constitutes the majority of a RISE session. This design reflects the RISE system’s primary goal: to serve as a structured educational tool for dementia caregivers. Rather than enabling open-ended or ad-hoc conversations, RISE provides targeted, evidence-based information tailored to specific caregiving concerns. Based on the caregiver’s choice of topic, the knowledge module follows a structured flow: a core presentation segment, a Q&A interaction, and a short quiz.

- During the presentation segment, the robot presents materials adapted from the REACH Caregiver Notebook in an easy-to-read format. During the presentation, the robot speaks, narrating the text displayed on the screen while gesturing and making eye contact to maintain engagement. The information is broken down into easily digestible parts, addressing the specific issues users provided at the module’s start. A screenshot of a presentation screen is shown in Figure 4b.
- In the Q&A segment, the user can ask clarifying questions and the robot will answer them. They can either type in their question via the tablet, or speak it using the tablet’s microphone. These questions can pertain to information they found confusing, topics not explicitly covered, or issues related to another module. The user speaks to the robot, which converts their speech to text and sends it to the embedded AI. All questions and answers are also displayed on the robot’s tablet screen. Figure 4c shows an example Q&A screen.
- After answering the user’s questions using the information from the notebook, the robot administers a brief quiz to test the user’s understanding of the material covered. The robot asks each question, displaying four answer choices on its tablet screen. Users select an answer, after which the robot informs them if their choice is correct and provides the correct answer with an explanation. The knowledge quiz module has five questions. After answering all five questions, the user receives a score and can either continue with the session or retake the quiz. This quiz reinforces the information presented and enhances user comprehension. Figure 4d shows an example quiz question.

Contents in the knowledge module are all generated using a RAG-AI model, to be detailed in the next section. Following the Knowledge Quiz, users return to the main menu, where they can choose to complete another Knowledge Module or select a Stress Management Activity to continue the RISE session. Users can complete as many Knowledge Modules as they wish, returning to the main menu after each one.

If the user does not wish to complete another Knowledge Module, they can proceed to a Stress Management Activity. Here, they can select from various activities derived from REACH, such as breathing exercises, guided imagery, and meditation, all aimed at reducing stress levels for dementia caregivers. The robot introduces these exercises and, once the user chooses an activity, guides them through a calming experience. Each activity is straightforward enough for users to follow and can be easily repeated independently at any time. After completing this activity, the RISE session comes to an end, and the robot thanks the user for participating.

### 3.2 The processing layers

Pepper’s physical interface, including speech synthesis, head and arm gestures, and basic dialog flow, is managed through its built-in software and standard development environment (Android Studio). As these features are handled automatically, this section focuses on our custom integration of a RAG pipeline that enables dynamic, content-grounded responses to caregiver queries.

To create a more personalized yet reliable information session, the RISE system utilizes a RAG system for all of its generated content. This allows the RISE system to pull from a certified knowledge base (the REACH Caregiver Notebook) and use an LLM (OpenAI’s GPT-4o) to generate the content. This backend system allows the RISE sessions to be generated differently for each user, allowing each session to be unique and cover the desired topics. Additionally, this feature also makes it possible to deliver the same content to the same user differently each time, reducing learner fatigue when a module is repeated.

**FIGURE 5:**
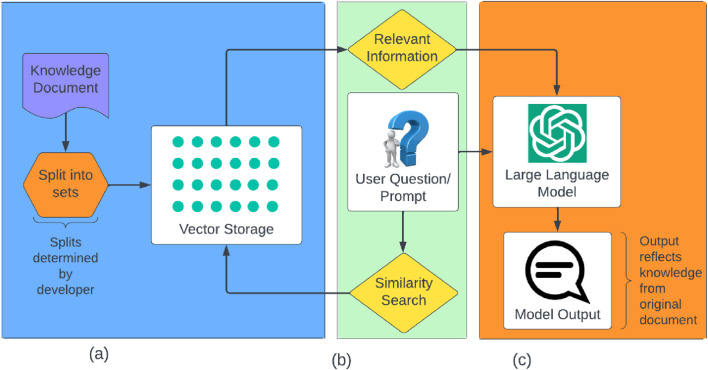
Flow of RAG-AI backend.

RAG systems are a newly emerging method of using generative AI while reducing hallucinations when generating content. To maintain the integrity of information, we use LangChain to validate all AI-generated outputs, ensuring they strictly adhere to the REACH caregiver resources and minimizing the risk of inaccuracies or biases. The system, illustrated in Figure 5, is built with three main components to produce an output to the user input: the vector storage, similarity search and information retrieval, and the large language model.

#### 3.2.1 Creating a knowledge base using vector storage

Based on the REACH Caregiver Notebook, we built a database from which information is pulled. For this purpose, we use Pinecone embeddings, a free-to-use, cloud-based storage system (Pinecone Systems, 2024).

We first converted the Caregiver Notebook from PDF to text by preserving its original structure, which is organized into 48 predefined chapters. These chapters—each focused on a specific behavioral or caregiver coping topic—served as the natural boundaries for segmenting the data. In other words, unlike a general-purpose RAG system where splits are arbitrary, we split the database based on the topics that were already present in the Notebook. By maintaining the structure of the original document, including its sectioning and topical groupings, we ensured that the RAG system could accurately retrieve context-specific information during each RISE session.

Once split, Pinecone vectorizes each piece of text and stores it in a vector storage database. Vectorization refers to the process of converting text from a string characters into numerical representations, called vectors, that capture the meaning and context of the text in a format that AI models can understand. These vectors enable the system to compare and retrieve semantically similar content, even if the exact words do not match.

This database is cloud-based, so we are able to access it at any time from anywhere. Such accessibility allows us to create multiple versions of the RISE system that all use the same database, which can be updated according to newer caregiving guidelines in the future.

Each module from the Caregiver Notebook spans approximately 5–10 pages. To accommodate the token limitations of the language model during response generation, we did not encode entire modules into the prompt. Instead, during preprocessing, each module was segmented into smaller, semantically coherent chunks based on natural boundaries such as headers, bullet lists, and paragraph transitions. These chunks typically ranged from 100 to 200 words. Each chunk was then vectorized using a sentence-level embedding model and stored in a vector database. During runtime, when a user poses a question, the system retrieves the top-k most semantically similar chunks using cosine similarity. These retrieved chunks are inserted into a prompt template and sent to the LLM to generate a relevant response. This approach allows the system to scale across long documents while ensuring that each AI response is grounded in focused, contextually relevant information.

In summary, this vector storage supports an intuitive, accessible, and searchable database that the RISE system can pull sources of information from for generating customized content.

#### 3.2.2 Retrieving information by similarity search

With the information now stored, the AI model can search it for relevant information to the user input. This is done first through a similarity search, which looks through the specified section(s) of the vector storage database for information relevant to the user input. For example, if the user asks a question on how to get their loved one to swallow properly while eating, the similarity search will look through the “Eating” chapter for information that pertains to swallowing.

Depending on content generation needs, we instruct RISE to search in different scopes via the AI prompt. Specifically, for the presentation, the RAG model only looks at the specified section that the user wants to cover, such as “Eating” or “Combativeness.” This limits the information of the presentation section to what is located in that specific section of the Caregiver Notebook.

However, in the Q&A section, a user might ask a question that is better answered in a different section. For example, if the user is completing the “Eating” module and asks, “How do I get my loved one to stop being aggressive while eating?”, the RAG system will look through the entire Notebook and find a better answer in the “Combativeness” module. This method allows the RAG system to generate the best possible answer to user inquiries based on its entire knowledge base, not just the single module the user is completing.

The most relevant information found is sent to the LLM as a part of the prompt. This ensures that the AI only uses the relevant, certified information from the database as its context when generating an output. In addition to the retrieved information, the original user prompt is fed into the LLM. This gives the LLM the required context to properly answer the user input, as well as the relevant information to generate an appropriate answer.

#### 3.2.3 Generating responses using a large language model

We use OpenAI’s GPT-4o model as the LLM provider, as it is one of the most robust systems that are commonly used and widely available. Other cloud-hosted LLMs, such as Google’s Gemini and Anthropic’s Claude, are possible alternatives and should work similarly.

When choosing the LLM provider, we have also considered hosting a local LLM, such as Meta’s Llama. There are several advantages of using a local LLM, including better privacy and data security. However, we currently use GPT-4o for two main reasons. First, hardware of the Pepper robot is rather limited. It is not powerful enough to host a local LLM. Primarily, Pepper does not have the dedicated GPU space to run a local LLM, and its CPU is fully taken up by its other processes (control, running apps, etc.). A workaround could be to host the local LLM on an auxiliary laptop near Pepper. This, however, would require a relatively powerful laptop given the size of the model and required processing speed. Second, compared to most LLMs that can be hosted locally, OpenAI’s GPT model has been performative, cost-effective, and sufficiently secure. Using a cloud-based LLM provider ensures the highest quality possible in generated responses. We use a standard Python server that utilizes OpenAI’s API services to call the AI model and generate content. Whenever we send a prompt to the AI, we give it detailed instructions on what it must do and the appropriate context from the Caregiver Notebook that was retrieved previously. Using this information, the LLM outputs the appropriate response (text for the presentation, a question for the Quiz, etc.). The prompts used for the three sections of the RISE session (presentation, Q&A, and quiz) are shown below in Table 2.

**TABLE 2:** Prompts used for each step of the RISE session.

Section	Prompt
Presentation	Slide 1 (Introduction): “Write a brief and short introduction to the [user-selected module] module from the ‘Introduction to Module’ section. Do not include the title of the module, and try to shorten the intro as much as possible.” Slide 2 (Basics and Summary): “Write the basic information that a caregiver should know from the [user-selected module] module from the ‘Basics and Summary’ section. Make it as concise, short, and easy to read as possible. Limit to 5 sentences, numbering each thought like: 1., 2., 3.” Slide 3 (Real Life Examples): “Quickly and efficiently list 2 common issues and solutions to them that involve the [user-selected module] module from the ‘Real Life Examples’ section, and number them like: 1., 2., etc. Limit it to four sentences in total response.” Slide 4 (Some Different Approaches): “Talk very briefly and efficiently at a 8th-grade reading level about 4 unique approaches and different outlooks on problems usually found in the [user-selected module] module from the ‘Some Different Approaches’ section. Number each new approach and follow with its name, like: ‘1. Approach Name’. Also, keep the explanations of each approach to 1 sentence only.”
Q&A	“Here is a user question: [question] and here is the relevant context to develop an answer: [context]. You will now answer questions based on the provided context. Limit your answers to no more than 2–4 sentences at a 8th-grade reading level. Summarize the information you find in a way that best answers the question.”
Review Quiz	For questions and correct answers: “Create 5 follow-up questions for the [user-selected module] module, make them challenging, but the answers obvious. Ensure they are only about and directly involved content in the [user-selected module] module. Each question will begin formatted like this ‘Q1:‘. Give the correct answers to these questions after the question is formatted to begin like so: ‘Answer:‘. Summarize the answers and keep them to less than 15 words. Do not have any other text other than questions and answers.” For incorrect answers: “Based on these questions and answers you created, generate 3 incorrect answers for each. Make the answers related to the scenario, but incorrect when compared to the correct answer. Summarize the answers and keep them to less than 15 words. Be sure to only include appropriate content.”

These prompts reflect minor adjustments made according to user feedback. They are also shortened for ease of reading.

The prompts used in each section of the RISE session were deliberately designed to support clear, concise, and structured interactions between the user and the AI system. Together, these prompt designs reflect an emphasis on user-centeredness, content fidelity, and accessibility, aligning with the overall RISE goal of delivering structured, evidence-based support through conversational AI.

- The prompt design for the presentation phase was inspired by principles of instructional scaffolding and layered content delivery. Each prompt sequentially introduces caregivers to the module topic using progressively deeper levels of information: starting with a high-level overview, moving into core facts, followed by common issues and solutions, and finally exploring alternative approaches. This structure mirrors best practices in caregiver education by delivering content in manageable chunks while reinforcing learning through repetition and variation.
- For the Q&A section, the prompt was guided by principles of semantic retrieval and simplification, encouraging the model to extract relevant information from the full knowledge base while summarizing it in a way that is accessible to caregivers with varying literacy or cognitive load. This phase emphasizes clarity, responsiveness, and a eighth-grade reading level to maximize understandability.
- The Knowledge Quiz consists of five AI-generated, multiple-choice questions based on the module. Each multiple-choice quiz question included one correct answer and four distractors. The correct answer was retrieved directly from the vector database containing the REACH Caregiver Notebook, ensuring that the information was evidence-based and clinically validated. To generate the incorrect answer choices (distractors), we used OpenAI’s GPT-4o model with controlled prompting. This approach ensured that distractors were plausible and contextually relevant without introducing misinformation.

The Review Quiz prompt was developed with active recall and formative assessment principles in mind. It instructs the model to generate challenging questions tied directly to the content of the selected module and to avoid obvious or trivial answers. By requiring answers to be summarized in fewer than 15 words, the system emphasizes concise reinforcement of key concepts without overwhelming the user. One issue we ran into when promoting the AI to generate the content was within the Review Quiz section. Because we use RAG and limit the AI to the specified knowledge base, it had difficulty coming up with incorrect answers for the questions. Often, all generated answers would be correct on a technical level, but only one was deemed “most correct.” To circumvent this issue, we call upon the AI model a second time after the questions and answers were generated. This time, we fed the AI the question and the answer it generated, and asked it to generate three incorrect answers. This time, however, it could not access the notebook, and instead was able to use its pre-trained knowledge base. This allowed the AI to generate answers that were noticeably incorrect when compared to the correct one. This is reflected in Table 2 under Review Quiz, where two prompts are listed: one to generate the questions and correct answers, and one to generate the incorrect answers.

A sample of AI-generated responses were reviewed by collaborators with dementia caregiving expertise to ensure clarity, accuracy, and appropriateness before adopting these prompts. The output is then properly formatted for displaying on the robot’s tablet screen. These outputs are also put through Pepper’s embedded text-to-speech program, so that Pepper can verbally present the information as well. Overall, this LLM model has provided robust and clear outputs that properly conveys the information from the Notebook.

#### 3.2.4 RAG evaluation

The RAG-AI generated content was systematically evaluated for its performance in content generation. We first curated a diverse set of 52 caregiver question-answer pairs as test cases. The questions cover across multiple modules (e.g., wandering, bathing, sleeping). The answers, written by domain experts, are considered the “ground truth.” The baseline model would be an LLM without any retrieval-based grounding. In this case, we simply used GPT-4o to generate answers without referring to the Caregiver Notebook. Using the Python library RAGAS (Es et al., 2025), we compared the quality of answers generated by RAG-AI with the baseline model on four different metrics:

1. Correctness: Measures how factually accurate and complete the generated answer is compared to the ground truth answer.
2. Relevancy: Assesses if the generated response directly and fully addresses the specific details of the user’s original question.
3. Faithfulness: Quantifies what proportion of the claims made in the final answer are explicitly supported by the information contained within the retrieved context documents (only for RAG).
4. Context Recall: Measures the percentage of critical facts from the correct ground truth answer that were successfully retrieved by the system’s database search and provided to the language model (only for RAG).

These metrics were calucated in specific was so they can be broken down into two main categories: metrics of generation (correctness and relevancy), and metrics of retrieval (faithfulness and context recall). RAGAS computed the correctness by combining the cosine similarity between the embeddings of the Answer and ground truth with an F1 score derived by asking an LLM to count the matching facts (true positives) between the answer and ground truth. This as a weighted average of two components: semantic similarity and factual alignment. Furthermore, the relevancy was done by using a “reverse question generation” technique. RAGAS asked an LLM to generate several hypothetical questions based only on the generated answer. Then, it calculated the cosine similarity between the vector embeddings of these generated questions and the embeddings of the original question, which leads to a higher similarity showing that the answer fits the question perfectly. Together, these two scores show the quality of the generation of the AI (i.e., how well the answer was generated). This could be done for the whole RAG-AI system, or any baseline generative AI model. Beyond this, for the faithfulness, RAGAS used an LLM to break the generated answer into individual statements, then verified if each statement could be inferred from the retrieved context. It then calculated a score by dividing the number of claims in the answer supported by the context by the total number of claims in the answer. Lastly, for the context recall, RAGAS used an LLM to break the ground truth answer into individual statements, then checked to see if each of these chunks could be found in the retrieved context. Similar to the faithfulness, RAGAS divided the number of statements in the ground truth found in the retrieved context by the total number of statements in the ground truth. Together, faithfulness and context recall show how well the RAG-AI system did at finding the relevant and correct chunks from the database to form its answers. Because both require the retrieved context for their calculations, these two metrics can only be computed for a RAG-AI system, and not any generic AI model.

Similar to existing studies (Pradhan et al., 2025; Darnell et al., 2024), we used an LLM as the judge. We opted to run the test with three different LLM models: OpenAI’s GPT-4o, Claude 4.5 Sonnet, and Llama 3.2. Since our RAG implementation used GPT-4o as part of the generation pipeline, it was natural to consider using GPT-4o as a judge as well. However, since it is possible that GPT would judge itself favorably, we also included Claude and Llama to evaluate GPT-4o to mitigate potential bias. Results are presented in Table 3.

**TABLE 3:** RAG evaluation results.

	GPT-4o		Claude 4.5 sonnet		Llama 3.2	
Metric	RAG-AI	Baseline	RAG-AI	Baseline	RAG-AI	Baseline
Correctness	0.914	0.289	0.874	0.329	0.892	0.584
Relevancy	0.945	0.921	0.916	0.887	0.927	0.779
Faithfulness	0.835	N/A	0.882	N/A	0.871	N/A
Context Recall	0.820	N/A	0.849	N/A	0.861	N/A

The RAG evaluation results revealed a few key patterns. First, the GPT-4o judge was the most lenient, giving the highest scores for both correctness and relevancy. This is likely because of an inherit bias as we also used GPT-4o to generate the content within the RAG system. As such, we expected the scores from this judge to be slightly higher. Another interesting point is that Llama 3.2, the smaller model, was also more lenient, especially with the baseline model. This is likely because this model is less sophisticated than the Claude model, and was therefore unable to perform as intense reasoning when judging the responses.

Regardless of the judge, the RAG approach adopted for RISE’s backend performed well across the board. Its correctness score (87% or higher) shows that the AI-generated responses were very close to the ground truth, justifying the idea that the system is able to generate valid answers. This is further supported by its relevancy score (92% or higher), indicating the AI stayed on topic a vast majority of the time. Both of the scores vastly outperform the baseline model among all judges, showing that the implementation of the RAG system allowed for the RISE system to use AI-generated content while staying both correct and relevant to the question.

Furthermore, the RAG-AI system had relatively high faithfulness and context recall, with Claude scoring those at 88% and 85%, respectively. This shows that the system was both able to retain the context it retrieved within its generated answer and that the context it retrieved was also present in the ground truth.

In summary, these results show that the prototype RISE system is not only functional, but does a great job at generating correct, relevant answers that stick to the database and are backed by facts. Its ability to stay on topic while using the verified database shows that the system can be trusted to provide useful information to informal dementia caregivers.

## 4 User evaluation methods

To evaluate the pre-prototype of the RISE system, we ran a two-phase study. The first phase included engineering students at our university who helped us identify usability issues and the acceptability of the system from a user standpoint, while the second included professional dementia caregivers researchers who helped evaluate the quality and accuracy of the generated content. This phased approach was chosen to separately address the system’s usability from an engineering perspective and the appropriateness and accuracy of the educational content from a clinical perspective, before advancing to testing with the more vulnerable population of actual dementia caregivers.

### 4.1 Usability measures

To assess user acceptance of the RISE system, we designed a caregiver feedback survey informed by established usability and human-robot interaction (HRI) assessment tools. Specifically, the survey design draws upon elements from the Questionnaire for User Interaction Satisfaction (QUIS) (Chin et al., 1988), the System Usability Scale (SUS) (Brooke, 1996), the Almere Model (Heerink et al., 2010), and the Computer System Usability Questionnaire (CSUQ) (Lewis, 1995). These instruments have been widely used to assess perceptions of system usability, social presence, trust, and overall satisfaction in HRI and health technology contexts. These constructs were chosen due to their relevance in understanding how users engage with novel assistive technologies—particularly those integrating AI and robotics.

The resulting survey consisted of 18 items, 12 of which were close-ended and are listed in Table 4. The remaining items—Questions 5, 9, 12, 14, 17, and 18—were open-ended and invited participants to elaborate on their experience with each RISE component or to suggest areas for improvement.

**TABLE 4:** User acceptance and usability survey questions.

Construct	Question	Format
UI: quantity	Q1. The amount of knowledge module choices was good	5-point Likert
IQ: presentation	Q2. The content in the AI-generated presentations was accurate (to my knowledge)	5-point Likert
PU: presentation	Q3. The content in the AI-generated presentations answered my questions	5-point Likert
UI: presentation	Q4. The layout of the AI-generated presentations was good	5-point Likert
	Q5. Do you have any other comments/concerns regarding the AI generated presentations?	Open-ended
IQ: Q&A session	Q6. The content in the AI-powered Q&A session was accurate (to my knowledge)	5-point Likert
PU: Q&A session	Q7. The content in the AI-powered Q&A session fully answered my questions	5-point Likert
UI: Q&A session	Q8. The layout of the AI-powered Q&A session was good	5-point Likert
	Q9. Do you have any other comments/concerns regarding the AI powered Q&A session?	Open-ended
IQ: knowledge quiz	Q10. The content in the AI-generated knowledge quiz was accurate (to my knowledge)	5-point Likert
UI: knowledge quiz	Q11. The layout of the AI-generated knowledge quiz was good	5-point Likert
	Q12. Do you have any other comments/concerns regarding the AI generated knowledge quiz?	Open-ended
UI: stress management	Q13. The layout for the stress management activity was good	5-point Likert
	Q14. Do you have any other comments/concerns regarding the stress management activity?	Open-ended
UI: ordering	Q15. The order of the activities in the RISE app was good	5-point Likert
WR	Q16. I would recommend the RISE app to a friend/fellow caregiver	5-point Likert

UI: user interface; IQ: information quality; PU: perceived usefulness; WR: willingness to recommend.

Each construct informed the development of our user survey, which was administered immediately following the RISE session. Survey items were adapted to assess user experience across four key domains: perceived usability, ease of interaction, system trustworthiness, and social presence. These domains are especially relevant to technologies like RISE, where the interface includes a humanoid robot delivering educational content through conversational interaction. All closed-ended questions were rated on a 5-point Likert scale (1 = strongly disagree to 5 = strongly agree). We included both Likert-scale questions and open-ended prompts to capture users’ impressions of system performance, usefulness, and comfort with interacting through speech. Additionally, several items focused on the perceived helpfulness of specific system components (e.g., the quiz, risk assessment, and robot behavior), enabling component-level feedback.

In addition to assessing the overall RISE system, we included questions specific to each RISE component (presentation, Q&A, quiz, and stress management activity) to evaluate system usability and user satisfaction. Open-ended items were incorporated to gather qualitative feedback on each section and to surface potential design issues not captured through structured questions.

This approach aligns with prior work on caregiver-facing AI tools, emphasizing simplicity, accessibility, and trust as central factors influencing acceptance. While the RISE survey does not reproduce any one instrument verbatim, it reflects the validated constructs present in the source models and ensures a caregiver-appropriate language and tone.

It should be noted that there is a distinction between usability and user acceptance. While the majority of the survey items assess core usability and satisfaction constructs, such as user interface (UI) and information quality (IQ), the overall instrument is explicitly designed to measure the preliminary user acceptance of this RISE prototype. This is achieved by using key predictive factors of technology adoption, including Perceived Usefulness (PU) and the Willingness to Recommend (WR). These function as a proxy for the behavioral intention to use, which is a critical element of established acceptance models like the Almere Model (Heerink et al., 2010). This approach ensures that we assess not only if the system is easy to use, but also if users find it valuable enough to consider adopting it for real-world support.

### 4.2 A two-phase user study

To evaluate both the technical functionality and content validity of the RISE system, we conducted a two-phase user evaluation. This phased approach was chosen to separately address the system’s usability from an engineering perspective, and the appropriateness and accuracy of the educational content from a clinical perspective. Phase 1 involved technically literate university students who interacted with the RISE prototype to identify user interface issues, system responsiveness, and general user experience. Phase 2 included expert reviewers with extensive experience in dementia caregiving interventions who evaluated the content accuracy, pedagogical structure, and real-world applicability of the system. By segmenting the evaluation in this way, we were able to gather targeted feedback from users with different expertise, ensuring both the functionality and the fidelity of the intervention were thoroughly assessed before broader deployment.

#### 4.2.1 Study phase 1

For the first phase, a convenience sample of university students were recruited to interact with the robot and complete a RISE session.

To recruit for this study phase, students were recruited through their professors (i.e., colleagues of the research team) during their class. Interested students contacted the graduate lead to set up a time for their session. There, they were explained the purpose of the study, exactly what was to be collected, and how long it should take. After agreeing to these points, the participant signed an informed consent form and began the study. Afterwards, they were thanked for their time. This study did not include any incentives. Furthermore, this study was supported by UTK IRB-23-07933-XP. The purpose of this student evaluation was to identify prototype concerns in how the system functioned in a controlled environment, minimizing the risk of causing distress to actual caregivers. First, they were asked to fill out a brief demographic survey. Next, they participated in a complete RISE session. This included the Risk Assessment, the Knowledge module (presentation, Q&A, and quiz), and a stress management activity. After completing the session, they were asked to fill out the usability survey described in subsection 4.1. Participants could leave additional comments on the presentation, Q&A session, knowledge quiz, stress management activity, AI-generated content, and general RISE app, respectively. Each survey question in the table was on the Likert scale, with 5 being the most positive and one the most negative. The survey results are discussed in detail later.

After completing the survey, the participant was asked to share any other opinions through a semi-structured interview. The researcher asked broad questions on the different points of the RISE session and asked the participants to share their opinions on the topic. By “semi-structured,” we mean that the interview did include a few prewritten questions, but the interviewer did not focus entirely on those. Instead, they were tasked with asking follow-up questions based on the participant’s responses. Therefore, the protocol for the interview was largely unstructured. It only sought to gather additional thoughts from participants that the research team might not have considered. To this end, the questions were extremely general (i.e., “What are your thoughts on using an AI in this capacity?”), and the interviewer came up with more questions in direct response to the participant (i.e., “Why do you think this?”). This semi-structured interview allowed participants to voice their own general thoughts, while also allowing research team members to dig into specific comments and learn more about the participants’ opinions of the RISE system. All participants were required to complete the interview.

#### 4.2.2 Study phase 2

To further evaluate the initial prototype of the RISE system, the two dementia caregiver training domain experts who had provided the information and materials to develop the app then reviewed the various aspects of the system. Due to the distance between collaborators, the most efficient method of review was to record a demonstration video and have the caregiver researchers review that. The video featured a complete RISE session, including starting the robot, the introduction/Risk Assessment Questionnaire, a Knowledge Module, and a Stress Management Activity. The researchers gave a comprehensive review of the system; specifically covering the design of the app, the content in the models, and the feasibility of using the robot.

## 5 Results

The results from both studies provided insights into this pre-prototype version of the RISE app. We received valuable feedback on the various UI and design aspects, as well as feasibility.

### 5.1 Phase 1 results

Five students were recruited for the study, with the demographics information summarized in Table 5. They were between 18 and 34 years old, split between male and female, and were either undergraduates, graduate students, or post graduations. These participants stated they were either “very” or “extremely” familiar with four types of technology: phones, tablets, computers, and robots.

**TABLE 5:** Participant demographics.

Demographic	Count
*Age*	
18-24	3
25-34	2
*Gender*	
Male	3
Female	2
*Education*	
Some College	1
College Graduate	2
Post-graduate	2
*Ethnicity*	
Caucasian	2
Asian	3

They had little to no experience with AI-based assistive technologies, but they did have experience with machine learning and simple AI tools. All participants believed a robot-based training program for dementia caregivers would be useful.

While the sample size is small, it is typical for a preliminary user study that is focused on collecting feedback (Zhao et al., 2025; Koutentakis et al., 2020). Additionally, their demographic composition spread across a diverse set of age and gender composition, and thus they are considered a somewhat representative sample of the technologically savvy users.

#### 5.1.1 Survey results

**FIGURE 6:**
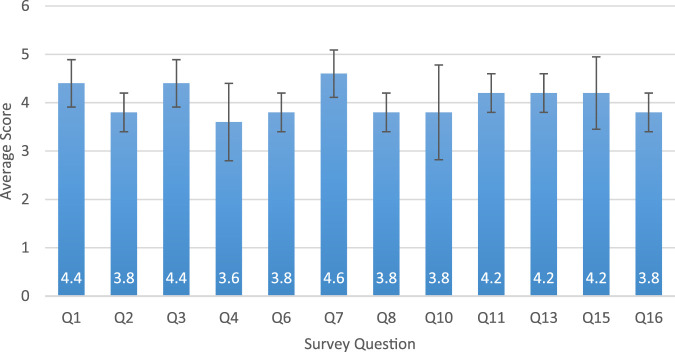
Average survey question responses from Phase 1 participants with standard deviation. The survey questions can be found in Table 4.

Results are shown in Figure 6. The first set of questions, Q1-5, revolves around the knowledge modules and the presentation. The participants were satisfied with the amount of knowledge modules (4.4 ± 0.49 for Q1) and the content within them (4.4 ± 0.49 for Q3). This shows an acceptance of RISE in delivering contents of the Caregiver Notebook by generating the presentation content.

Although the score of Q2 is relatively lower (3.8 ± 0.4 for Q2), participants indicated they did not distrust the content, however they had no contextual backing to certify the accuracy of the content because they were not dementia caregivers or experts. They therefore were more likely to give it a three or four out of 5. Participants were less satisfied with the layout of the AI-generated presentation slides (3.6 ± 0.8 for Q4). Several noted that the text seemed too dense or the robot spoke too quickly. This was further reflected in Q5 (an open-ended question), where participants were able to leave written comments on the presentation section. One participant wrote “The robot spoke too fast,” which is something also mentioned by the expert reviews. Another noted “Presentations are text dense, and could use pictures to enhance the visuals.” Given current constraints of Pepper’s interface, we were unable to implement a visualized presentation using AI generated images or videos. Since our current content validation has been limited to text, the RISE system still uses a text-based presentation. However, we adjusted the prompt to generate shorter text presentations using easy-to-understand language. Visual presentations could be a value-adding next step for system development and validation.

The next batch of questions, Q6-9, covered the Q&A portion of the Knowledge Module. This section was a strong point in each study session, with each participant saying they enjoyed the layout (3.8 ± 0.4 for Q8) and being able to speak to the robot. They found that it fully answered their questions (4.6 ± 0.49 for Q7), the layout was pleasing (3.8 ± 0.4 for Q8), and the content was accurate to their knowledge (3.8 ± 0.4 for Q6). Furthermore, the only comment left on Q9 was that the microphone took a second to load, however, this was not an issue with the RISE App, but rather with the robot, and only happened once.

Q10-12 reviewed the knowledge quiz. Once again, Figure 6 shows high results from the participants, with all being satisfied with the content (3.8 ± 0.98 for Q10) and layout (4.2 ± 0.4 for Q11) of the quiz. Furthermore, the only comment left on Q12 was “Some questions were too obvious, but this seems to be the purpose.” This observation is correct, as the review questions are meant to be easy and only serve to highlight various key ideas from the chosen module.

The questions on the stress management section, Q13-Q14, were also scored highly (4.2 ± 0.4 for Q13, and no comments on the open-ended Q14). The participants were satisfied with the layout of the activities, and none had any major comments. Overall, they found the stress management activities currently satisfactory, with satisfying layouts and engaging activities.

The last set of questions Q15-16, were on the RISE app and robot in general. As seen in Figure 6, the participants were satisfied with the order of the activities (4.2 ± 0.75 for Q15) and would recommend the session to someone else (3.8 ± 0.4 for Q16). However, they did note that they would only recommend the RISE app once the prototype has been developed a bit more, as the current version could use the adjustments previously described. For example, one comment regarding the AI generation was the extra time it took to generate. In general, it takes a few moments for Pepper to process the signals, the RISE app to connect to the Wifi and the Cloud-based servers, and for GPT-4o to generate the content. While many of these sources of latency are outside of our control, we can add UI aspects to help alleviate user frustration. These adjustments are discussed later on.

#### 5.1.2 Interview results

During the interview portion of the study session, participants were free to comment on various aspects of the RISE session. One repeated comment was a need for a visual cue when the AI model was generating content. In the current version of the RISE app, there is no additional change when a user starts the AI generation process, which as previously noted can take a moment. Therefore, participants encouraged adding an additional feature to notify the user when AI begins loading, which would also prevent excessive button tapping that could crash the app.

Additionally, during the interview participants pointed out that if the buttons changed colors when pressed then it would give more feedback to the users. This would be especially helpful during the Risk Assessment so users knew that they clicked the right button.

The last point brought up during the interview was a few bugs in the robot’s speech. For example, sometimes the robot would stop speaking and not finish what it was saying. Other than those few issues, the interviews brought up no other major concerns or comments on the RISE system.

### 5.2 Phase 2 results

The goal of Phase 2 was to evaluate the clinical relevance, content accuracy, and real-world applicability of the RISE system from the perspective of subject matter experts in dementia caregiving. While Phase 1 focused on identifying usability issues and refining interface design, Phase 2 was intended to validate that the AI-generated educational content aligned with best practices and retained the integrity of evidence-based caregiver interventions.

After reviewing the demo video, the caregiver researchers were pleased with the current state of the RISE system. However, they did have several concerns about the UI, robot behavior, and content. Concerns fell into three categories: robot speech, session instructions, and terminology.

#### 5.2.1 Robot speech

One concern the researchers had was the way the Pepper robot spoke. First, they were concerned with the speech speed, stating that sometimes Pepper was hard to follow. This was an easy adjustment to make and has already been done. The on board text-to-speech function was adjusted to set the speaking speed to 80%.

However, they also had an issue with the lack of pauses when giving the presentation. Because we use GPT-4o to generate the slides, it is difficult to control the spacing of voice lines in these presentations. More specifically, between the numbered points there should be a pause in the speech. However, the response we receive from the AI model does not contain this pause, so the robot does not when presenting the slides. Based on this feedback, we adjusted Pepper’s speech rate to 80% of the default and modified instructional phrasing to avoid confusion. Ongoing work focuses on enhancing speech segmentation, either by instructing the AI to insert explicit pauses or post-processing AI responses with timed speech breaks. By instructing the AI to provide responses with explicit breaks, we are able to cause pauses in the robot’s speech. This creates a slower, more natural speaking rhythm.

#### 5.2.2 Session instructions and terminology

The second critique the researchers had was the instructions the robot gave between session segments. For another example, when introducing the Risk Assessment, the robot said “Please take time to fill out this Risk Assessment Quiz”. However, the researchers pointed out that the Risk Assessment was not a quiz, and should not be considered as such. This is an example of an incorrect instruction that could be fixed by editing the scripted speech. We, therefore, changed the instruction to “Please take a moment to fill out this Risk Assessment Questionnaire, checking any issues you may be having in your caregiving journey.” We made similar adjustments to instructions throughout the RISE session so each was clear, concise, and correct.

The researchers expressed concern about some of the terminology used in the RISE session. For example, the term “module” to describe the knowledge sections might be misconstrued by some users. They pointed out that this is not an issue at the moment, but we are nonetheless exploring different options for this and other terms throughout the RISE session.

### 5.3 Insights from user feedback and areas for improvement

Addressing user feedback offers an opportunity to elevate RISE’s engagement and usability. Enhancements such as adding visual cues during content loading, adjusting robot speech speed and cadence, and refining the user interface could transform user experiences by reducing frustration and making interactions more intuitive. For example, introducing visual loading indicators not only prevents unnecessary user actions but also reinforces confidence in the system’s responsiveness. Similarly, tailoring the presentation layout to include concise text and optional visual aids could cater to diverse learning preferences, expanding accessibility across different user demographics. These iterative improvements, informed by feedback, would create a seamless and adaptive experience, increasing caregiver satisfaction and fostering greater trust in RISE’s capabilities.

The results from the Phase 1 and Phase 2 studies highlighted three key areas for improvement: 1) UI design, 2) robot behavior, and 3) AI generation. Both student participants and caregiver researchers consistently identified these areas as critical to enhancing the system’s usability and effectiveness, making them the primary focus for future development.

#### 5.3.1 UI design

A repeated point in the study sessions and throughout the expert feedback was the need for adjustments to various UI elements. The first was the AI-generated presentation. Several participants noted that the slides were very text-heavy, and some visual aids would make them more appealing. However, as previously stated, the 240 necessary images would be a huge strain on the Pepper robot’s memory. We are exploring other solutions, such as cloud storage, that would allow us to store the images on a separate server. The main issue with this solution is that it could cause an increase in load times for the session, as that is another server that must be called. By this, we mean that a second instance of an AI or cloud server must be connected, which would increase the load time for the modules. However, it is still a possible solution to an issue several people have had.

Another design change that was requested is some form of loading icon to be used to indicate that the AI is generating content. This would enable the user to know that something is happening and alleviate possible frustration. As of now, the primary solution is to add a video-based icon when the button to generate content is pressed. So long as this icon is present, the user will know that the AI system is working, and they need to wait for it to continue the session.

Lastly, reviewers suggested color changes to provide better user feedback and make the design more appealing. Primarily, some users suggested that buttons change color when pressed to provide more feedback to the user. The primary issue with this is how Android Studio defines button layouts and colors. To change the color by pressing a button, the layout itself would have to be redefined. This would exponentially increase the number of changes happening with each button press. One alternative solution to this is to change the buttons entirely. For example, instead of the “yes” or “no” buttons for the Risk Assessment portion, we could have checkboxes for each problem statement. This way users can identify which statements they have marked and we do not have to define hundreds of new button layouts. Such design changes will become a major avenue of future development for the RISE program.

#### 5.3.2 Robot behavior

When it comes to the robot itself, both the caregiver researchers and the student reviewers found that the robot spoke too fast and without pausing. First, the speaking speed has been adjusted and is currently at 80%. Adjustments to this speaking speed are easy, as the Pepper SDK controls it through a single variable. As such, this speed will be tested with further iterations of the RISE app, in future studies. Changes to the robot’s pitch and pronunciations are more difficult, however, as those are not controlled within the SDK. Lowering the speaking speed does lower pitch, but it is not controlled. Therefore, we will have to explore solutions on how to ensure proper pronunciation and an easy-to-understand pitch.

Secondly, we are working on adding pauses between lines during the knowledge presentations. This is more difficult, as previously discussed because the responses from the AI model are input directly as speech lines and do not contain pauses. We are testing a few methods to add pauses to the GPT-4o response, such as requesting them in the initial prompt. We are also looking into manually adding them once we receive a response from the AI. Such changes are doable, but require preliminary testing. Therefore, we are experimenting with each of them simultaneously, manipulating both the AI model and the robot’s speech function. We are seeking solutions to this challenge. As for the movement of the robot, all respondents seem satisfied. No participant or expert saw the movements of the robots as excessive, which is ideal because the default movements were used. The robot movements are a product of the SDK used to program the RISE app, and therefore are already adjusted to optimal levels. For this reason, we have no plans to adjust this aspect of the robot’s behavior.

#### 5.3.3 AI generation

The most novel and important aspect of the RISE app is the use of AI to generate content. Since all of the content generated comes directly from the REACH Caregiver Notebook, we expect high accuracy and reliability of the information given. However, one major technical and usability concern was brought up by users and researchers.

The primary concern was the length of time required for the AI to generate the presentation slides and quiz questions. While this is primarily a limitation of Wi-Fi connectivity or server lag, there are methods we can use to decrease the generation time. For example, some clever prompt engineering would allow us to reduce the information the AI needs, which could increase the response time. Altogether, AI response time is difficult for us to control, but there are emerging solutions. One area of concern from both user groups was the latency in AI response during presentation and quiz generation. This delay led to uncertainty about whether the system was working. While some delay is expected due to the real-time nature of AI content generation, we plan to mitigate its impact through improved UI design—such as adding animated loading indicators or auditory cues—and optimizing prompt construction to reduce token complexity. Additionally, we are exploring pre-caching content for frequently used modules to further reduce perceived wait times.

#### 5.3.4 Overall results and study limitations

Overall, this preliminary study shows exceptional acceptance of the RISE system among tech-savvy students and dementia caregiving experts. Not only this, but the study has provided us with ample avenues to continue our research and ways to improve the RISE system. While it was not ideal to run the study with just students instead of actual dementia caregivers, it still provided valuable insight. We learned a lot about how the UI could change and become more user-friendly, as well as ways to improve the general feasibility of the system. However, this learning is not a substitute for running studies with caregivers, and we are already in the process of doing this. We plan to bring the robot to care facilities where informal dementia caregivers often congregate or visit, such as a local neuroscience clinic. Nonetheless, this preliminary study with this population has provided us with ample information and insights on how to continue to improve the RISE system.

A primary limitation of this pilot evaluation is the limited sample size and participant demographics. Phase 1 participants were university students without personal experience in dementia caregiving. While this group allowed us to evaluate usability and technical robustness in a low-risk environment, their ability to assess clinical content accuracy was limited. Furthermore, while the phase 2 reviewers were highly experienced researchers and had dementia caregiving experience, there were only two of them. Future iterations of this study will include testing with unpaid dementia caregivers to ensure ecological validity and better assess the system’s clinical relevance.

The two-phase design was a deliberate risk-mitigation strategy to ensure both the technical usability (via Phase 1) and the clinical fidelity (via Phase 2) were assessed before testing RISE with a more vulnerable population (i.e., informal dementia caregivers). The Phase 1 findings were more straightforward, giving insights into the usability of the system and areas of improvement for the UI. The use of tech-savvy university students gave us insights into the general acceptance of the RISE system and where it meets user standards (as well as where it fails to do so). This population gave us these insights without putting risks onto the more vulnerable population of informal dementia caregivers, who could be unintentionally harmed by improper AI generation or technology that is difficult to use. Moreover, the Phase 2 results, specifically the expert reviews, were crucial for validating the quality of the AI generated content and the consistency of the responses against the REACH database. These experts provided domain-specific feedback on terminology (e.g., changing “quiz” to “questionnaire” for the Risk Assessment) and robot pacing. Furthermore, the content validation done through the stress test assessed the generated answers for their clinical relevance and safety. While more rigorous testing of the AI outputs must be done in the future, that is not the focus of this study. This study, sought to give us insights into the overall user acceptance and feasibility of the RISE system. The results from both phases do just that, with Phase 1 highlighting which aspects are accepted by users and what areas can be improved, while Phase 2 gives an informal review of the validity of the AI generated content.

## 6 Discussion and future directions

### 6.1 Comparison with existing caregiver technologies

Existing technology-based caregiver interventions are often delivered through mobile apps, websites, or printed handbooks (Elliott et al., 2010; Gitlin et al., 2015; Burgio and Wynn, 2021). These tools are often standalone or adapted versions of structured programs (like REACH) that typically lack the personalized, two-way interaction provided by the RISE system.While these tools provide valuable information, they are typically static, limited in personalization, and require the caregiver to initiate and interpret the information independently. Several studies have demonstrated benefits from structured educational interventions delivered in person by a human coach, such as REACH (Wisniewski et al., 2003; Czaja et al., 2018; Nichols et al., 2017; Martindale-Adams et al., 2017; Nichols et al., 2016; Belle et al., 2006), but their scalability remains constrained.

In contrast, the RISE system uniquely combines a social robot with AI-generated, evidence-based content to deliver a highly interactive and personalized caregiving experience. Unlike conventional mobile applications, RISE dynamically tailors sessions to individual caregiver concerns via natural dialogue and gesture, simulating a human educator. This hybrid of embodied social presence and generative AI sets RISE apart from prior tools.

Due to the encouraging results from the two-phase evaluation, not only is it apparent that the RISE system is well accepted (albeit with a few necessary improvements), but there are also signs of how RISE positively compares to existing caregiver tools and interventions. Due to the way RISE is built, we know that it differs in ways such as personalization, interactivity, and accessibility. A full comparison list is shown below in Table 6.

**TABLE 6:** RISE compared to existing technology-focused caregiver tools.

Feature	RISE system	Existing technology-focused caregiver tools
Technology	AI-powered social robot (Pepper) with interactive features	Mobile apps, websites, printed guides
Content Source	Evidence-based REACH Caregiver Notebook, delivered via generative AI	Standardized content, often lacking personalization
Personalization	Dynamic personalization through Risk Assessment and tailored knowledge modules	Limited personalization (e.g., user-selected topics in apps)
Interactivity	Interactive presentations, Q&A sessions, and quizzes facilitated by a social robot	Text-based tools or pre-recorded videos
Stress Management	Integrated stress reduction activities (e.g., guided breathing, meditation)	Standalone apps or external resources
Accessibility	Available through robot interface in clinical or caregiving environments	Often requires smartphone or computer access
User Experience	Voice, gesture, and screen-based interaction to simulate human communication	Static UI in apps and guides
Feedback Mechanism	AI-powered Q&A sessions and real-time quizzes for knowledge retention	Limited feedback mechanisms (e.g., app surveys)
Ethical Safeguards	Fully localized AI processing, opt-out options for sensitive questions, disclaimers	General privacy settings in apps

Looking at this comparison, the main ways RISE differs from existing technology-focused caregiver tools are in its two-way interactions and its content personalization. First, the RISE system interacts with users in a way not yet seen with other dementia caregiving tools, other than those delivered by a human coach. Oftentimes, caregivers use tools through an app on a tablet/smartphone or a computer website. These tools are static, respond very little to user input, and are limited in their ability to provide feedback. The RISE system, however, offers a unique form of interaction through the social robot and generative AI. This allows it to respond to virtually any user input that it receives, and it can provide custom responses to a user’s question or quiz responses. The social robot also makes the session more engaging, since its movements and voice are often more appealing than a static tablet screen. Overall, the RISE system is far more interactive than existing caregiver tools.

In addition to its interactive capabilities, the RISE system also excels in its ability to provide personalized content using generative AI. Existing caregiver tools often use prebuilt modules with no user customizations, as well as premade quizzes for review and lack of details on specific issues. However, the RISE system can take into consideration a user’s specific issues and create a personalized session to cover specific topics. This level of customization is often only available in one-on-one counseling sessions with professional dementia caregiver trainers, which as previously stated is difficult to disseminate. Therefore, its ability to create personalized information sessions for each user puts RISE a step above other existing technology-focused caregiver tools.

While a formal SWOT analysis on direct and indirect competitors was not performed for this preliminary evaluation, the comparison framework in Table 6 serves an equivalent function by directly contrasting key features, such as Interactivity, Personalization, User Experience, and Content Source. This analysis highlights the functional gaps in existing static tools, demonstrates the strengths of the RISE prototype, and suggests opportunities for advancing connected health technologies. Future developmental phases will incorporate a formal competitive analysis to inform the inclusion of new functionalities and mitigate potential threats to real-world deployment. This, however, is outside of the scope of this preliminary study. Instead, this overview justifies the scientific merits of the RISE system and its contributions to the field.

While the RISE system could just be a simple chatbot that uses an LLM, the choice of an AI-powered SAR is justified by the plethora of advantages of embodiment and social presence in delivering sensitive health-related guidance, especially to high-stress populations like dementia caregivers. Unlike a static screen or a simple chatbot, the humanoid robot Pepper engages users through lifelike gestures, voice, and empathetic interactions that actively maintain engagement. Studies indicate social robots are generally perceived as more engaging than mobile apps or web pages. This embodied interaction provides a higher level of engagement and simulates a user more closely than any text-based application. This is crucial for RISE’s ability to deliver structured behavioral interventions, such as the REACH components, which traditionally rely on personalized, person-delivered coaching and empathetic guidance. In essence, the social robot serves as a compelling, low-friction interface that maximizes user comfort and learning reinforcement in clinical, community, or home settings.

### 6.2 Implementation strategies for real-world deployment

To enable RISE’s adoption in real-world healthcare settings, strategic partnerships will be essential. While we are already working with a senior center, further collaborating with hospitals, community health centers, and caregiver support networks can provide critical pathways for deployment. For instance, placing RISE systems in hospital waiting rooms or caregiver resource centers could create convenient access points for caregivers to interact with the robot while awaiting consultations. Similarly, partnerships with organizations like the Alzheimer’s Association or other caregiver advocacy groups could promote the system’s adoption and expand its reach within underserved communities.

Addressing regulatory and ethical considerations will be crucial for successful implementation. Since RISE leverages AI and robotics in sensitive caregiving contexts, compliance with healthcare data privacy regulations, such as HIPAA in the U.S., will ensure the protection of user information. Ethical safeguards must also include transparency in AI-driven decisions, disclaimers about the system’s limitations as a non-clinical tool, and protocols for managing user distress. While running a local LLM could help reduce these concerns, as previously mentioned such models are too computationally intensive for the Pepper robot or even a remote system. However, the cloud based LLM’s, like the GPT model RISE currently uses, do have multiple safety factors already. Establishing regular audits and updates to the AI content will further align RISE with evolving best practices in healthcare.

Scalability and sustainability will require a focus on cost-effectiveness and long-term maintenance. Leveraging the existing manufacturing scale of the Pepper robots, coupled with cloud-based updates for the RISE app, can reduce initial and ongoing costs. Additionally, modular designs for software updates will allow continuous improvement without requiring hardware replacements. Establishing a subscription or sponsorship model through healthcare providers or caregiver networks could subsidize costs for end-users, making the system more accessible. By addressing these considerations, RISE could become a transformative, scalable solution for dementia caregiver support.

### 6.3 Ethical considerations

Given that dementia caregivers represent a vulnerable population often experiencing high levels of stress and emotional burden, several ethical safeguards were implemented in this study and the RISE system design. First, all study procedures were reviewed and approved by the Institutional Review Board at the University of Tennessee, with special attention to participant privacy and data protection protocols.

For data security and privacy, all interactions between users and the RISE system are conducted locally, with no personal information stored on external servers. The AI-generated content is created using only the pre-approved REACH Caregiver Notebook materials, ensuring that all advice and information provided to caregivers is evidence-based and clinically validated. To maintain transparency, users are informed that they are interacting with AI-generated content delivered through a robotic interface.

The system is designed to be supportive rather than prescriptive, explicitly stating that it does not replace professional medical advice or emergency services. Clear disclaimers are provided at the beginning of each session, and users are reminded to consult healthcare professionals for specific medical concerns. The stress management activities are based on well-established, low-risk techniques from the REACH program. For the validation study, we deliberately chose to begin with student participants rather than actual caregivers to identify and address any potential usability issues or technical problems that might cause frustration or distress to caregivers. The expert review phase included experienced dementia researchers who could evaluate the system’s appropriateness for the target population.

Future implementations of RISE in healthcare settings will require additional ethical considerations, including:

- Regular content reviews by healthcare professionals
- Clear protocols for handling crisis situations
- Ongoing assessment of the system’s impact on caregiver wellbeing
- Mechanisms for user feedback and continuous improvement
- Equity considerations regarding access and technological literacy

These ethical safeguards were designed to ensure that RISE provides beneficial support while protecting user wellbeing and maintaining high standards of care. To avoid causing unnecessary distress or frustration to vulnerable caregivers, we deliberately conducted initial testing with a healthy, tech-literate population to refine system usability. Following IRB approval, we are now recruiting informal dementia caregivers for the next phase of testing to ensure the system’s content relevance, emotional appropriateness, and overall benefit in real-world contexts.

### 6.4 Limitations and future work

One concern that was raised with the research team is the availability of the Pepper robot. The robots we have were produced in 2020 and are no longer in production; however, they can still be purchased from RobotLab and programmed. In fact, the RISE system was developed and tested within the past year, showing that continually progressive systems can still be created for this slightly outdated robot. Furthermore, the Pepper robot was used because there are still no social robots that are as expressive or as easy to use as it. This means the RISE system can use novel and sophisticated AI tools while also utilizing the extremely engaging and well-tested Pepper robot model.

We are also considering changes to the selected LLM. As stated before, we use GPT-4o-mini as it is sophisticated, fast, and cheap. We also use this cloud based model as running a local LLM is not feasible on the Pepper robot, as well as to intensive and requires additional hardware for running remotely. However, we can still stick to a cloud-based, pretrained LLM moving forward. In fact, OpenAI is constantly rolling out new models, and it is easy to update which one we are using since the RAG-AI backend is entirely on the cloud. Should OpenAI (or any other company) come out with a faster, more sophisticated, yet still cost effective model, we can easily update RISE. This allows us to keep our system on the front-end of AI development, putting to use the newest advancements of AI technology.

Additionally, the technical evaluation could be done more comprehensively, with room for improvement. For example, further research could address improvements of the technical metrics, such as a fine-tuning the RISE model to achieve higher scores across the board. Such improvements could help the RAG-AI backend generate better answers and retrieve more relevant context from the database. We will plan to run more explicit testing with all three parts of the RISE system in a future paper focused on an intense technological evaluation. This preliminary study of the RISE system provides crucial insights, but it does have limitations that inform our plans for future work. A primary limitation is the participant demographics of the Phase 1 study, which are limited to tech-savvy university students. While this group was idea for assessing the system’s usability in a low-risk environment, their ability to assess the clinical accuracy and appropriateness of the system was limited. This approach, however, was a conscious choice designed to protect the wellbeing of the target population. Our goal here was to refine the system’s interface and usability to address any potential technical issues that would later cause frustration for informal dementia caregivers. By first testing this system with tech-savvy students, we were able to identify and fix these issues before introducing them to the target population.

Moving forward, our research will focus on full-scale user testing with the informal dementia caregivers. This future study is crucial to ensuring the RISE system’s validity and assess its user acceptance in a real-world setting. We plan to recruit informal dementia caregivers at care facilities, such as neuroscience clinics and senior centers, where they often visit. This will allow us to gather valuable feedback on how the RISE system interacts with the target population and how well accepted it is.

## 7 Conclusion

This paper presented the development and evaluation of RISE, a novel AI-powered robotic system designed to support dementia caregivers through automated education and stress management. The system combines generative AI technology with a social robot interface to deliver personalized content from evidence-based REACH protocols. We conducted a two-phase evaluation study, gathering feedback from student participants for technical concerns and expert dementia researchers for content, to assess the system’s usability, acceptance, and potential for real-world implementation.

Overall, the reviews we have received from the study participants and dementia caregiver researchers have provided critical feedback and multiple points for future development. Our findings demonstrate both the technical viability and user acceptance of an AI-powered robotic system for caregiver support, with survey scores averaging above 3.8 out of 5 across all evaluated dimensions. The survey results, interview responses, and expert feedback all indicate that users accept the RISE system as a viable educational tool. Most understand that this is only a prototype, thus, more testing and adjustments are needed. Moreover, each reviewer found the RISE system to be an acceptable way to support dementia caregivers.

Based on the reviews, we were informed what aspects to improve. Changes to the UI design, the robot’s speech patterns, and the AI generation time are important focus points for us as we continue to develop the novel RISE system. We believe the RISE system can become an ideal program for bringing information to caregivers, thus improving both the quality of life and mental health of caregivers and care receivers.

The RISE system shows promise as a practical, accessible solution for caregiver education and support. By combining AI-driven personalization with the engaging presence of a social robot, we have created a platform that can be scaled up to meet the growing needs of the caregiving community, while maintaining the quality and reliability of evidence-based care protocols. By fostering a more personalized, interactive, and supportive caregiving experience, RISE represents a crucial step forward in transforming connected health technologies. With further development, it has the potential to empower caregivers, reduce their stress, and enhance the quality of life for millions of families affected by dementia.

## Data Availability

The raw data supporting the conclusions of this article will be made available by the authors, without undue reservation.

## Footnotes

1 While this model of the Pepper robot is no longer in production, the robot is still available to purchase through Robotlab. We expect newer models of social robots, with functionalities similar to Pepper, to reach similar results
