# Supplementary material for: Transforming dementia caregiver support with AI-powered social robotics
Source: Front Robot AI. 2026 Jan 27;12:1704313. doi: 10.3389/frobt.2025.1704313 (PMC12886016; doi:10.3389/frobt.2025.1704313)
Supplement: Supplementary file 1 [file DataSheet2.pdf]

Appendix B: Sample RAG Stress Test Outputs

The following examples are drawn from a stress test conducted on the RAG-AI Q&A pipeline used in the RISE system. These illustrate typical responses the system generated based on queries related to common dementia caregiving challenges.

1. **Test Question:** What should I do if my loved one smokes?
   RAG-AI Response:

   If your loved one smokes, consider the following steps to ensure their safety and well-being.
   - **Decide on Smoking Rules:** Determine if you want your loved one to stop smoking or if you're comfortable with them smoking under monitored conditions
   - **Reduce Smoking Triggers:** Remove cigarettes, lighters, matches, and ashtrays that could remind them of smoking. Limit beverages like cola or coffee that they associate with smoking, offering juice instead. Be mindful of trigger times, such as after meals or during phone calls.
   - **Create a Safe Smoking Environment:** If smoking cannot be avoided, designate a safe place for smoking, like the kitchen or balcony. Use large, deep ashtrays with water for added safety, and ensure smoking is done away from flammable materials
   - **Support and Monitoring:** Establish a smoking schedule to monitor smoking breaks. Ask friends and family to support the new smoking rules.
   - **Safety Measures:** Install working smoke detectors and fire extinguishers. Post emergency numbers visibly.
   - **Health Support:** Discuss with your loved one's doctor about nicotine replacement therapies like patches or gum, or medications that might help reduce the desire to smoke.
   - **Awareness of Risks:** Understand that smoking is a leading cause of house fires and that unsupervised smoking can increase this risk.

   By taking these steps, you can help manage your loved one's smoking in a way that prioritizes their health and safety.

2. **Test Question:** What should I do if I miss a scheduled doctor's appointment?
   RAG-AI Response:

   If you miss a scheduled doctor's appointment, the materials do not provide specific instructions for this scenario. However, general guidance would be to contact the doctor's office as soon as possible to inform them of the missed appointment and to reschedule for the next available time. It's also important to discuss any immediate concerns or changes in your loved one's condition that may need prompt attention.

3. **Test Question:** What should I do if I feel that I don't have enough time for myself?
   RAG-AI Response:

   If you feel that you don't have enough time for yourself, consider these steps:
   - **Ask for Help:** Reach out to friends or family members to stay with your loved one, allowing you some time to rest or go out.
   - **Utilize Tools for Short Breaks:** Use a childproof doorknob on your bathroom door for privacy, set a timer for short breaks, or use baby monitors, plug-in intercoms, or walkie-talkies to stay in touch with your loved one from another room.

- **Adult Day Care:** Consider taking your loved one to adult day care for personal time. This also benefits your loved one by providing social interaction and activities
- **Communicate Clearly:** When leaving the room, use visual aids like a drawing of a clock to show when you'll return, aligning it with a real clock to help your loved one understand.
- **Healthy Lifestyle:** Engage in exercise, eat balanced meals, and ensure you get enough rest. These activities can help manage stress and improve your well-being.
- **Enjoy Simple Pleasures:** Even if it's just for 15 minutes, take time out for activities you enjoy. This is crucial for your well-being.
