# Supplementary material for: Transforming dementia caregiver support with AI-powered social robotics
Source: Front Robot AI. 2026 Jan 27;12:1704313. doi: 10.3389/frobt.2025.1704313 (PMC12886016; doi:10.3389/frobt.2025.1704313)
Supplement: Supplementary file 2 [file DataSheet1.pdf]

APPENDIX A: KEY CONTENT IMPLEMENTED IN RISE

Appendix A.1: Risk Assessment

The RISE Risk Assessment consists of 23 items, adapted from the REACH VA caregiver screening tool. Caregivers are asked to answer “Yes” or “No” to the following issues:

1. Sleeping during the day and keeping you up at night
2. Showing little interest in daily activity
3. Having problems with bathing or refusing to bathe
4. Arguing, irritability, complaining, or aggressive
5. Trouble remembering recent or significant past events
6. Losing or misplacing things
7. Difficulty concentrating on a task
8. Appearing sad or depressed or crying
9. Appearing anxious or worried
10. Having problems with dressing or refusing to get dressed
11. Refusing to eat or eating too much
12. Believing someone is stealing from them
13. Seeing people or things that are not there
14. Accidents of the bowel or bladder
15. Waking you or other family members up at night
16. Asking the same question over and over
17. Inappropriate sexual behavior
18. Following you everywhere or getting upset if you leave the room
19. Getting upset when friends or family visit
20. Wandering outside the house
21. Problems with driving such as getting lost or not paying attention
22. Problems with dangerous items, such as a gun or knife
23. Safety concerns about your loved one smoking

Appendix A.2: Knowledge Modules

The RISE system includes 48 Knowledge Modules based on the REACH Caregiver Notebook. These are grouped into two categories: 30 behavioral topics and 18 caregiver coping and self-care topics.

Behavioral Topics

1. Activities
2. Bathing
3. Combativeness
4. Communicating with a Person with Dementia
5. Confusion

6. Dental Care
7. Depression
8. Dressing
9. Driving
10. Early-stage Dementia
11. Eating
12. Environment
13. Feelings
14. Grief
15. Hallucinations and Delusions
16. Holidays
17. Hospitalization
18. Incontinence
19. Medications
20. Nutrition
21. Repeated Questions
22. Safety
23. Sexuality
24. Shadowing
25. Sleeping
26. Sundowning
27. Telling the Patient and Others
28. Traveling
29. Visiting
30. Wandering

Caregiver Coping and Self-Care Topics

1. Addressing Problems
2. Adult Day Care
3. Asking for Help
4. Communicating with Health Care Providers
5. Depression
6. Early-Stage Dementia
7. Feelings
8. Financial and Legal Issues
9. Getting Help
10. Grief
11. Healthy Lifestyle

12. Holidays
13. Lifting and Moving
14. Making New Friends
15. Mood Management
16. Sexuality
17. Stress Management
18. Visiting
